# Long-term macular atrophy growth in neovascular age-related macular degeneration: influential factors and role of genetic variants

**DOI:** 10.1038/s41433-025-03723-3

**Published:** 2025-03-10

**Authors:** Brice Nguedia Vofo, Yahel Shwartz, Yaacov Cnaany, Shlomit Jaskoll, Adi Kramer, Sarah Elbaz-Hayoun, Batya Rinsky, Michelle Grunin, Liran Tiosano, Itay Chowers

**Affiliations:** 1 Department of Ophthalmology, Hadassah-Hebrew University Medical Center, and the Faculty of Medicine, The Hebrew University of Jerusalem, Jerusalem, Israel; 2 Department of Military Medicine and “Tzameret”, Faculty of Medicine, Hebrew University of Jerusalem, Jerusalem, Israel

**Keywords:** Risk factors, Medical imaging

## Abstract

**Objectives:**

This retrospective cohort study aimed to assess the long-term growth and associated risk factors of macular atrophy (MA) in eyes with neovascular age-related macular degeneration (nAMD) treated with intravitreal anti-vascular endothelial growth factor (anti-VEGF) compounds.

**Methods:**

Two hundred and six patients initiating anti-VEGF therapy were followed for 8 years using a treat-and-extend protocol. The study analysed correlations between MA growth (by square root transformation measured in infrared images) and clinical parameters, and genetic variants for AMD in the complement and lipid pathways and the ARMS2 gene.

**Results:**

Seventy-six patients (*n* = 92 eyes) were included, with a mean age of 73.9 ± 7.9 years. Eyes received an average of 7.1 ± 3.2 anti-VEGF injections per year. The prevalence of MA increased from 28.3% at baseline to 78.3% at 8 years, exhibiting an average annual growth rate of 0.25 ± 0.22 mm. Correlations were found between MA growth and size, and number of atrophic foci at baseline, and the common ARMS2 variant. Eyes with subretinal fluid (SRF) at baseline showed less foveal atrophy at 8 years compared to those with IRF or both IRF and SRF. No correlation was observed between MA growth and genetic variants in the complement and lipid pathways.

**Conclusion:**

Most eyes with nAMD under 8 years of anti-VEGF therapy developed MA, with significant growth. Correlations with baseline MA characteristics and the ARMS2 variant were identified. Further investigation is needed to understand the potential role of complement as a therapeutic target for preventing macular atrophy in nAMD-affected eyes.

## Introduction

The introduction of anti-vascular endothelial growth factor (anti-VEGF) therapy in the management of neovascular age-related macular degeneration (nAMD) has revolutionize the treatment landscape, providing significant visual gains in patients with nAMD [1]. However, in the long term, these patients continue to require monthly injections and the initial visual gains are often lost due to the development of macula atrophy (MA) and scar [2–4].

Presence of intraretinal fluid (IRF) [5, 6], reticular pseudodrusen [5, 6], type 2 and 3 macula neovascularization [7], thin subfoveal choroidal thickness, posterior vitreous detachment [6], epiretinal membrane [8], pre-existing geographic atrophy (GA) prior to the development of choroidal neovascularization [9], ^(p15)^ GA in the fellow-eye [8], and distance of the GA lesion from the fovea [8], are factors that have been associated with MA development and progression in nAMD. Such new atrophy of the retinal pigment epithelium (RPE) and choriocapillaris in nAMD is often observed within or near the neovascular lesion [8, 10]. The association between anti-VEGF treatment and the development and growth of MA remains a matter of controversy [11, 12].

The genetic component of AMD has been well-established, with over 50 loci identified through genome-wide association studies [13, 14]. Variants in complement factor H (CFH) and age-related maculopathy susceptibility 2 (ARMS2) genes have been shown to confer the greatest increased risk for AMD [15]. ARMS2 variant, in particular, has been associated with higher rates of MA progression in some studies [16, 17]; however, their association in nAMD eyes remains unclear. Lipid metabolism has also been implicated in AMD pathogenesis, with studies describing an association between serum lipid levels and AMD risk [18, 19]. Lipid metabolism genes such as apolipoprotein E (APOE), cholesteryl ester transfer protein (CETP) and hepatic lipase (LIPC) gene have also been associated with AMD risk [20–22]. However, their role in the development and progression of MA is still uncertain.

The recent approval of pegcetacoplan and avacincaptad pegol, complement inhibition-based compounds, for the treatment of GA, has brought forth new possibilities. Intravitreal injections of these compound have shown an approximate 20% reduction in GA progression [23, 24]. This breakthrough raises intriguing questions about the role of complement and of genetic variants in the complement cascade in the development and progression of MA in nAMD. Additionally, there is a growing interest in the potential efficacy of complement inhibition in addressing MA in eyes affected by nAMD.

This study aims to investigate the rate of growth of MA over 8 years in nAMD eyes under treatment with anti-VEGF and its association with genetic risk factors in the complement and lipid pathways and with ARMS2 genetic variants.

## Methods

### Patients

This retrospective study comprised patients initiating anti-VEGF treatment at the Hadassah Medical Center, Jerusalem, from January 2006 to December 2011. Hadassah’s Institutional Ethics Review Board approval was obtained, and patients provided informed consent after explanation of the nature and possible consequences of the study (Prior to blood collection). Inclusion criteria involved consecutive cases of nAMD treated with anti-VEGF for a minimum of 96 months (8 years). Exclusion criteria encompassed prior photodynamic therapy, retinal comorbidity, significant cataracts, and ocular surgeries other than uncomplicated cataract surgery. Bilateral cases were included if they met criteria. nAMD diagnosis was based on ophthalmoscopy, optical coherence tomography (OCT), and fluorescein angiography according to Age-Related Eye Disease Study (AREDS) criteria.

### Data collection

Demographic and clinical data were retrospectively collected from electronic medical records. Anti-VEGF treatment followed a structured protocol, initially involving three monthly 1.25 mg bevacizumab injections, followed by a “treat-and-extend” regimen (TER). OCT findings guided treatment timing, considering intraretinal fluid (IRF) and subretinal fluid (SRF) persistence or recurrence. Treatment adjustments occurred based on guidelines, switching between bevacizumab, ranibizumab, or aflibercept as needed. MNV type was determined using SD-OCT and FA as described previously [25].

### OCT and infrared reflectance image analysis and annotation

**Fig. 1:**
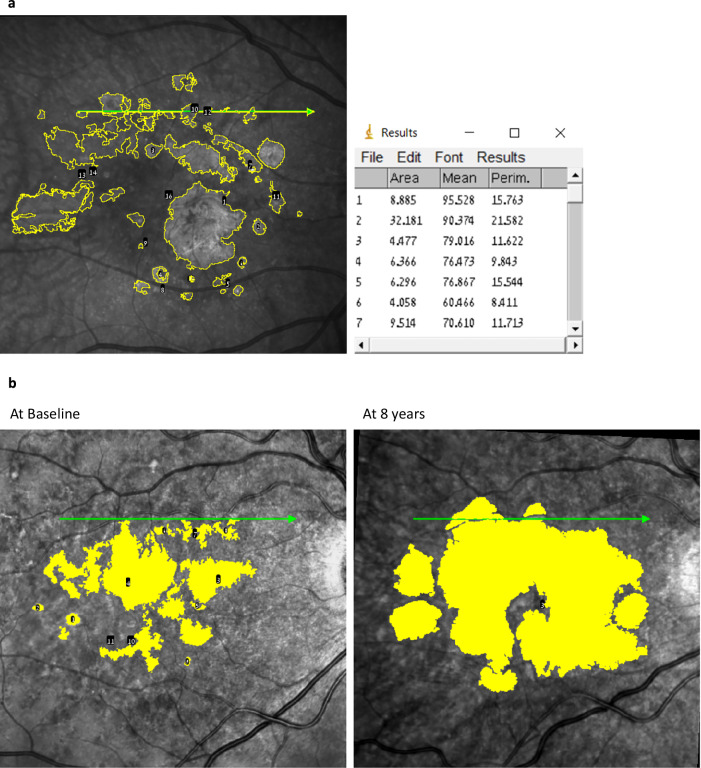
Delineation of atrophic foci using image analysis. **a** IR and SD-OCT images with color enhancement were analyzed using ImageJ to identify atrophic areas, which are highlighted in yellow. **b** Progression of atrophic foci from baseline to 8 years is depicted, illustrating the growth of the affected regions. The table on the right summarizes the measured MA foci and the corresponding area of each focus.

MA size assessments were conducted at the Hadassah Ophthalmology Reading Center following standardized protocols. Atrophy, defined as complete RPE and outer retina atrophy (hypo-reflective regions), was initially verified on OCT using the reflective and tracking reference tools in the Heidelberg interface [26, 27]. This verification ensured that structural atrophy identified on OCT was accurately reflected in the corresponding IR images. Once validated, the OCT and infrared reflectance (IR) images were exported in TIFF format, and analysis was performed using ImageJ software version 1.53 (National Institutes of Health, Bethesda, MD, USA) as previously described [28]. IR images were manually cropped to include only the macular region (central 6 mm × 6 mm) to ensure consistency and eliminate non-relevant peripheral areas. The scale of all cropped IR images was standardized to the same resolution (e.g., 1 pixel = 20 μm) to enable accurate comparisons. Experienced graders annotated MA lesions on IR images (Fig. 1a, b). Atrophy, defined as complete RPE and outer retina atrophy (hypo-reflective regions), was determined on OCT as previously described [26]. Lesions were highlighted using a standardized brush size and colour in ImageJ. Discrepancies between graders were resolved by a senior grader or retinal specialist to ensure consistency and accuracy. Annotated IR scans were saved for future measurements, and the total MA area and perimeter were calculated. To account for baseline atrophy areas, a square root transformation (SQRT) was applied in the analysis of atrophy growth rates, as described by Yehoshua et al. [29] They found that the rate of macula atrophy enlargement varied with different baseline atrophy areas. However, when the SQRT of lesion area measurements was used as a measure of lesion size, the enlargement rate no longer correlated with baseline size. For example, for baseline and follow-up MA area measurements of 1 mm² and 4 mm², respectively, the SQRT values used in the analysis would be √1 mm^2^=1 mm and √4 mm^2^ = 2 mm, respectively.

### Assessing spatial correlation between MNV and MA area

The location of MNV at baseline was marked on B-scan OCT, and the corresponding line was marked on the IR image. After eight years, recorded atrophy areas were overlaid on the baseline MNV location.

### Genotyping

Genotyping was performed on an exome chip platform as part of a previous study [13], and genotyping data on common variants was extracted. The genetic risk score (GRS) was calculated for each subject (*i* = 1,…,*n*) based on a set of k single nucleotide polymorphisms (SNPs) considered, where the number of risk alleles was encoded as 0, 1, or 2.

The GRS for each individual was generated using the formula: ${\rm{GRS}}={\sum }_{i=1}^{n}{G}_{i}{W}_{i}$

Where Gi represents the genotype of variant i, coded as 0, 1, or 2 based on the number of risk alleles (0, 1 or 2). W_i_ represents the effect size of variant *i* (natural logarithm of the fully conditioned odds ratio (OR) of the minor alleles of variant i), based on a prior genome-wide association study where marginal genetic effects of corresponding SNPs were estimated. These weights reflect the approximate impact of each SNP on AMD risk, based on ORs [13]. This method considers the varying contributions of genetic variants from different pathways to an overall genetic risk [30].

We created two distinct genetic risk scores, one for complement pathway, involving 19 related variants, and another score centred on the lipid pathway, comprising 7 associated variants. Variants were assigned to pathways using a functional enrichment analysis conducted by Fritsche and colleagues with the INRICH tool [31]. Supplementary Table 1 provides a list of these variants and their corresponding pathways.

### Statistical analysis

SPSS version 25.0 was used for analyses. Group comparisons employed paired Student *t*-tests or Wilcoxon signed-rank tests. Categorical parameter prevalence was compared using Fisher’s exact test, and correlations were assessed using Spearman’s correlation coefficient. In the analysis of factors affecting macula atrophy growth, only one eye (right eye) was included in cases of bilaterality, to avoid bias stemming from subject-specific factors. Statistical significance was set at *p* < 0.05.

## Results

### Patient characteristics

Between January 2006 and December 2011, a total of 276 eyes from 206 patients started intravitreal bevacizumab treatment for newly diagnosed nAMD. Of the 276 eligible eyes, 92 eyes (33.3%) in 76 patients (36.9%) were followed for a period of at least 8 years and were included in this study (dropout rate = 66.7%). The average age was 73.9 ± 7.9 years, with 42 (55.3%) of them being female. On average, eyes received 7.1 ± 3.2 (mean ± SD) anti-VEGF injections per year. Type 1 MNV was the most prevalent (44.6%), followed by type 2 (41.3%) and type 3 (14.1%; Table 1). Supplementary Table 2 presents a comparison of baseline demographics and OCT findings between eyes that completed the 8-year follow-up and those lost to follow-up at any point before the 8-year mark.

**Table 1:** Summary of patient characteristics at baseline (*N* = 76).

Patient characteristics	*N* (%)
Gender	
Female	42 (55.3%)
Male	34 (44.7%)
Age^a^	73.9 ± 7.9
<70	21 (27.6%)
70–80	39 (51.3%)
>80	16 (21.1%)
Smoking	
Yes	15 (19.7%)
No	38 (50.0%)
Past	16 (21.1%)
Unknown	7 (9.2%)
Hypertension	
Yes	45 (59.2%)
No	28 (36.8%)
Unknown	3 (4.0%)
Diabetes mellitus	
Yes	16 (21.1%)
No	57 (75.0%)
Unknown	3 (3.9%)
Coronary artery disease	
Yes	21 (27.6%)
No	52 (68.4%)
Unknown	3 (4.0%)

### Prevalence and growth of MA

**Fig. 2:**
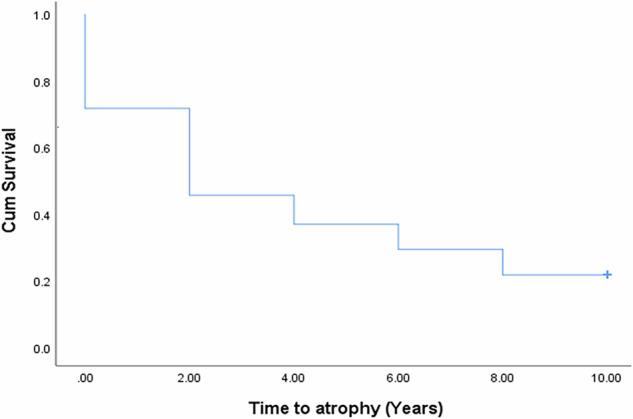
Survival curve to the development of MA in nAMD eyes from baseline.

**Fig. 3:**
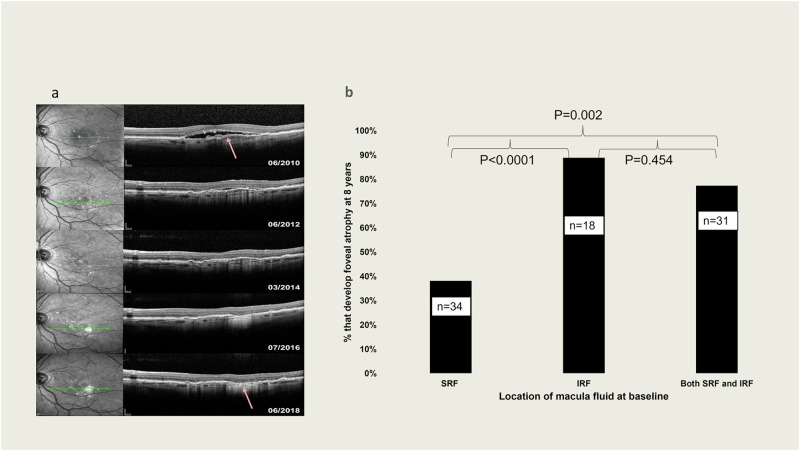
Macular Atrophy (MA) development and foveal atrophy risk based on baseline features. **a** Example of MA development after 8 years of follow-up in area that had macula neovascularization at baseline. The arrow indicates point the original MNV. **b** Percentage of eyes that developed foveal atrophy based on location of macula fluid at baseline. According to the analysis of SD-OCT. Groups compared by Fisher exact test.

MA prevalence increased from 28.3% at baseline to 78.3% at 8 years (*p* < 0.0001). The mean SQRT of atrophy demonstrated a substantial rise from 0.36 ± 0.72 mm at baseline to 2.01 ± 1.83 mm at the final visit (*p* < 0.0001), corresponding to an average growth rate of 0.25 ± 0.22 mm/year (0.84 ± 1.31 mm²/year). Eyes with IRF at baseline experience an average annual growth rate of MA of 0.363 ± 0.185 mm/year vs 0.141 ± 0.205 mm/year in eyes with no IRF at baseline (*p* < 0.0001). However, no difference in rate of MA progression was seen to be associated with presence of SRF at baseline. The median survival time for atrophy development was estimated at 2 years (95% confidence interval: 0.83-3.17 years; Fig. 2). Furthermore, the median number of atrophic foci per eye increased from 0 (interquartile range: 0–1) at baseline to 4 (interquartile range: 0–9) at 8 years (*p* < 0.0001). Notably, the growth of atrophy was positively correlated with the size and number of atrophic areas observed at baseline (Spearman’s *ρ* = 0.469 and 0.449, *p* < 0.0001 for both, respectively). Conversely, no associations were found between the growth of MA and age, the number of intravitreal injections, or the type of MNV (Table 2). Interestingly, in all eyes where MA developed, it encompassed the area of MNV observed at baseline (Fig. 3a).

**Table 2:** Correlation between initial measurements and subsequent changes in size and quantity of atrophic foci, along with various risk factors.

	Growth in atrophy (SQRT)		Increase in number of atrophic foci		Size of atrophy at baseline (SQRT)		Number of atrophic foci at baseline	
	Spearman’s Correlation Coefficient	*P* value	Spearman’s Correlation Coefficient	*P* value	Spearman’s Correlation Coefficient	*P* value	Spearman’s Correlation Coefficient	*P* value
Age	−0.185	0.149	−0.153	0.236	−0.084	0.515	0.014	0.915
Mean number of injections per year	−0.225	0.070	−0.116	0.354	−0.062	0.620	−0.090	0.472
Genetic risk score for complement pathway	−0.127	0.321	−0.033	0.795	-0.308	0.232	−0.267	0.350
Genetic risk score for lipid pathway	−0.124	0.323	−0.042	0.741	−0.305	0.132	−0.266	0.130
ARMS2	0.283	0.021	0.216	0.081	0.177	0.155	0.204	0.076
Number of atrophic foci at baseline	0.449	<0.0001	0.151	0.225	0.947	<0.0001	NA	
Increase in number of atrophic foci	0.723	<0.0001	NA		0.176	0.156	0.151	0.225
Time to atrophy (years)	−0.768	<0.0001	−0.526	<0.0001	NA		NA	
Size of atrophy at baseline	0.469	<0.0001	0.176	0.156	NA		0.947	<0.0001

*SQRT* square root transformation, *NA* not applicable, *ARMS2* age-related maculopathy susceptibility 2.

### Macula fluid and atrophy

Eyes were categorized based on the presence of SRF, IRF, or both SRF and IRF at baseline. The development of MA at 8 years was assessed in relation to these fluid characteristics. The analysis showed that fewer eyes with SRF at baseline (38.2%, *n* = 34) developed foveal atrophy at 8 years, compared to eyes with IRF (88.9%, *n* = 18) or both IRF and SRF (77.4%, *n* = 31) at baseline (*p* < 0.0001 and *p* = 0.002, respectively; Fig. 3b).

### Genetic risk factors and atrophy

The presence of major genetic risk variants for AMD in the complement cascade and the lipid metabolism pathway (see Supplementary Table 1 for list of variants) in this cohort of patients was correlated with the development and progression of MA. Though we found no correlation between the complement and lipid pathways genetic risk scores and development or growth of MA, the ARMS2 variant correlated with the growth of MA (Spearman’s *ρ* 0.283, *p* = 0.021; Table 2). Eyes with IRF at baseline had a higher mean number of ARMS2 risk alleles than eyes with no IRF at baseline (1.109 ± 0.795 vs 0.696 ± 0.832, *p* = 0.044). However, the statistical power for excluding association between the complement and lipid genetic risk scores and the rate of MA progression in this cohort of nAMD eyes was only 38.6%, and 19.7%, respectively.

### Multiple regression

A multiple linear regression model was calculated to predict change in MA (SQRT), based on the size of atrophy at baseline, number of atrophic foci at baseline, presence of intraretinal fluid only at baseline, and the ARMS2 variant; factors which were found to be associated with change in MA (SQRT) in univariate analysis. A significant regression equation was found (F(3,62) = 9.032, *p* < 0.0001), with *R*^2^ = 0.304. The size of atrophy at baseline and risk alleles for the ARMS2 variant accounted for a significant amount of unique variance (*p* = 0.003 and 0.032, respectively). Over 8 years of follow-up, the size of MA (SQRT) increases by 0.466 mm (95% confidence interval: 0.389–1.759 mm), for every mm increase in baseline MA (SQRT). Likewise, the size of MA at 8 years (SQRT) increases by 0.241 mm (95% confidence interval: 0.045–0.992 mm) for every additional ARMS2 risk allele.

## Discussion

This study investigated the growth patterns and associated factors of MA in nAMD eyes treated with anti-VEGF injections for a span of 8 years. The research unveiled crucial insights into the progression of MA in nAMD. Initially, we identified a connection between the size and quantity of atrophic foci at the study’s commencement and the subsequent rate of MA expansion. This indicates that the atrophy extent at the first clinic exam could potentially forecast its progression in nAMD patients undergoing anti-VEGF therapy. Furthermore, MA consistently expanded into the zone previously affected by MNV. This discovery underscores the intertwined relationship between neovascularization and atrophy development in nAMD eyes.

Eyes exhibiting SRF at the study’s commencement exhibited a diminished probability of MA development compared to eyes with IRF or both IRF and SRF. This differentiation underscores how the localization of fluid could differentially impact subsequent atrophy development in nAMD eyes.

The ARMS2 variant correlated with faster progression of MA at 8 years. Patients carrying ARMS2 risk alleles were more likely to have intraretinal fluid at baseline. We found no discernible correlation between the genetic risk scores for the complement and lipid pathways and the presence of MA and the number of atrophic foci at baseline. The rate of atrophy growth and increase in number of atrophic foci over 8 years in this cohort was also not associated with the lipid and complement genetic risk scores. These findings corroborate with that of a recent post-hoc analysis of an Age-Related Eye Disease Study 2 cohort which found that the ARMS2 variant was associated with faster progression of GA in non-neovascular AMD [32]. The European Eye Epidemiology consortium also investigated the phenotypic course and spectrum of AMD for the risk haplotype at the ARMS2 in a large European population study [26]. They concluded that the ARMS2 variant acts as a strong catalyst of AMD progression once early signs are present and carried a higher risk for MNV in comparison to the phenotypic spectrum of complement genes. This study finding indicate that the ARMS2 variant also drive MA development in nAMD eyes under treatment with anti-VEGF compounds.

Blodi et al.‘s post-hoc analysis of the Marina study revealed a higher incidence of MA in eyes treated with ranibizumab compared to sham-treated eyes, indicating that anti-VEGF injections could indeed contribute to MA development [33]. Interestingly, the MNV in the sham eyes may have offered some protection against the development of atrophy. However, once atrophy occurred, the rate of its enlargement was similar in both groups. Although our study was not sham-controlled and thus cannot verify this, we observed at 8 years follow-up that macula atrophy had involved the area of the initial MNV in all eyes. Limited number of studies have investigated the long-term development and growth of MA in nAMD eyes. In this study, we examined the mean SQRT atrophy growth per year, which was measured as 0.25 ± 0.22 mm (0.83 mm²/year). Our findings align with a study by Eng et al. [34] which reported an average cRORA growth rate (±SD) of 0.60 ± 1.69 mm²/year in nAMD eyes over a 2-year period. However, Eng et al. did not perform a square root transformation to account for baseline atrophy size, as described by Yehoshua et al. [29] Another study by Xu et al. [7] described atrophy growth in nAMD eyes, reporting growth rates of 0.54 mm/year (95% CI, 0.27–0.82 mm/year), 1.43 mm/year (95% CI, 0.94–1.91 mm/year), 0.80 mm/year (95% CI, 0.64–0.96 mm/year), and 1.23 mm/year (95% CI, 0.97–1.49 mm/year) in types 1, 2, 3, and mixed form neovascularization (MNV) in 94 eyes with nAMD, respectively. These data indicated that the odds of atrophy progression were lower in type 1 MNV compared to the other types. On average, the reported growth rates in Xu et al.‘s study were higher than those in our study, and MNV type was not identified as a factor associated with atrophy progression in our findings. The differences in follow-up duration between the two studies (96 months in our study vs. 28.5 months in Xu et al.‘s study) may contribute to the disparate findings. Accordingly, the additional observations of atrophy growth eventually involving the area of MNV is a finding that is more likely to be apparent over time requiring long-term follow-up and could bring about this disparity in our results.

Our study also demonstrated that eyes with SRF at baseline were less likely to develop MA compared to eyes with IRF or both IRF and SRF. This finding aligns with the results of the Fluid Study, a 24-month multicentre, randomized single-masked non-inferiority clinical trial [35]. The Fluid Study investigated the hypothesis of tolerating some SRF (less than 200 μm at the foveal centre) in patients with nAMD undergoing anti-VEGF treatment. The study concluded that tolerating SRF did not result in higher visual acuity loss compared to a more intensive treatment approach aiming for complete resolution of macula fluid. Our findings in this study are consistent with those of the Fluid Study, as we demonstrate that SRF is less associated with MA and, consequently, visual acuity loss in nAMD eyes undergoing anti-VEGF treatment.

Surprisingly, we found no correlation between the complement and lipid genetic risk scores, and the rate of MA progression in this cohort of nAMD eyes. We had previously reported an inverse correlation between the number of risk alleles in *CFH* and/or *C3* and the change in macular thickness measured at 8 and 10 years in nAMD eyes under anti-VEGF therapy [2]. However, in nAMD eyes, other factors, such as location of fluid and MNV, and dynamics of resolution of fluid, may come into play affecting the rate of MA growth. Additionally, this study did not have enough power to exclude a type 2 error, thus, we cannot exclude correlation of the lipid and complement genetic risk scores with MA growth.

Caveats to this study include its retrospective design that introduces inherent limitations and potential biases. Additionally, the relatively small number of cases during the follow-up period should be considered when interpreting the results. We also did not analyze the visual acuity. Yet, a notable strength of our study lies in its relatively long-term follow-up duration of a consecutive group of patients, and the availability of genotyping data, which enhances the robustness and depth of our findings. Additionally, visual acuity is not considered a primary outcome in trials assessing therapies for MA.

In summary, our findings indicate that the majority of eyes with nAMD undergoing anti-VEGF therapy for 8 years exhibited a substantial MA. The growth of atrophy was significantly associated with the initial MA characteristics and presence of ARMS2 risk alleles. Furthermore, baseline IRF and not SRF was associated with more atrophy. Importantly, our study did not observe any correlation between atrophy growth and major risk alleles in complement genes that are associated with the development of AMD. These findings warrant further research to uncover the underlying mechanisms driving MA in nAMD. Particularly, the potential role of complement activation in MA in nAMD should be carefully evaluated and confirmed prior to the application of anti-complement-based therapies in this clinical scenario.

## Summary

### What was known before

- Several factors have been identified as potential contributors to macular atrophy in neovascular age-related macular degeneration (nAMD) over short and medium terms. However, there is a lack of comprehensive studies examining these factors over a longer term.
- While the influence of genetic variants in the ARMS2, complement, and lipid pathways on the progression of atrophy in dry AMD is known, their specific impact on macular atrophy progression in nAMD has remained unclear.

### What this study adds

- This study reveals that, over the long-term, eyes developing macular atrophy typically do so in areas where initial macular neovascularization occurred. Furthermore, it was observed that eyes with baseline intraretinal fluid are more prone to develop foveal atrophy.
- Within this cohort, the ARMS2 genetic variant, but not variants in the complement and lipid pathways, was found to be associated with the development of macular atrophy in eyes affected by nAMD.

## Supplementary information

Supplemental Table 1

Supplemental Table 2

## Data Availability

The datasets used and/or analyzed during the current study are available from the corresponding author on reasonable request.
