# Supplementary material for: Long-term macular atrophy growth in neovascular age-related macular degeneration: influential factors and role of genetic variants
Source: Eye (Lond). 2025 Mar 10;39(9):1717–23. doi: 10.1038/s41433-025-03723-3 (PMC12130187; doi:10.1038/s41433-025-03723-3)
Supplement: Supplementary file 1 — Supplemental Table 1 [file 41433_2025_3723_MOESM1_ESM.docx]

**Supplementary Table 1: Independent AMD risk variants, chromosome positions and effect sizes (Odds Ratios) analyzed in each participant.**

| **Pathway** | **Locus name** | **Index variant** | **Chromosomal position** | **Minor allele/Major allele** | **Odds ratio** |
| --- | --- | --- | --- | --- | --- |
|  | CFH | rs10922109 | 1:196,704,632 | C/A | … |
| Complement | CFH | rs570618 | 1:196,657,064 | G/T | 1.65 |
| pathway | CFH | rs121913059 | 1:196,716,375 | C/T | 33.2 |
|  | CFH | rs148553336 | 1:196,613,173 | T/C | 0.31 |
|  | CFH | rs187328863 | 1:196,380,158 | C/T | 1.43 |
|  | CFH (CFHR3/CFHR1) ^*^ | rs61818925 | 1:196,815,450 | G/T | 1.18 |
|  | CFH | rs35292876 | 1:196,706,642 | C/T | 1.55 |
|  | CFH | rs191281603 | 1:196,958,651 | C/G | 0.39 |
|  | *CFI* | rs10033900 | 4:110,659,067 | C/T | 1.15 |
|  | *CFI* | rs141853578 | 4:110,685,820 | C/T | 3.64 |
|  | *C9* | rs62358361 | 5:39,327,888 | G/T | 1.8 |
|  | *C2/CFB/SKIV2L* | rs116503776 | 6:31,930,462 | G/A | 0.57 |
|  | *C2/CFB/SKIV2L* | rs144629244 | 6:31,946,792 | G/A | 1.39 |
|  | *C2/CFB/SKIV2L (PBX2)* ^*^ | rs114254831 | 6:32,155,581 | A/G | 1.13 |
|  | *C2/CFB/SKIV2L* | rs181705462 | 6:31,947,027 | G/T | 1.55 |
|  | *C3* | rs2230199 | 19:6,718,387 | C/G | 1.43 |
|  | *C3* | rs147859257 | 19:6,718,146 | T/G | 2.86 |
|  | *C3 (NRTN/FUT6)* ^*^ | rs12019136 | 19:5,835,677 | G/A | 0.71 |
|  | *TMEM97/VTN* | rs11080055 | 17:26,649,724 | C/A | 0.91 |
|  | *APOE* | rs429358 | 19:45,411,941 | T/C | 0.7 |
| Lipid  pathway | *APOE(EXOC3L2/MARK4)* ^*^ | rs73036519 | 19:45,748,362 | G/C | 0.91 |
|  | *CETP* | rs5817082 | 16:56,997,349 | C/CA | 0.84 |
|  | *CETP* | rs17231506 | 16:56,994,528 | C/T | 1.16 |
|  | *ABCA1* | rs2740488 | 9:107,661,742 | A/C | 0.9 |
|  | *LIPC* | rs2043085 | 15:58,680,954 | T/C | 0.87 |
|  | *LIPC* | rs2070895 | 15:58,723,939 | G/A | 0.87 |
|  | *ARMS2* | rs3750846 | 10:124,215,565 | T/C | 2.81 |

^*^The peak of this independent association signal is located closer to genes in parenthesis than to the locus-defining genes. Chromosome and chromosomal position according to the NCBI RefSeq hg19 human genome reference assembly.
