# Supplementary material for: Long-term macular atrophy growth in neovascular age-related macular degeneration: influential factors and role of genetic variants
Source: Eye (Lond). 2025 Mar 10;39(9):1717–23. doi: 10.1038/s41433-025-03723-3 (PMC12130187; doi:10.1038/s41433-025-03723-3)
Supplement: Supplementary file 2 — Supplemental Table 2 [file 41433_2025_3723_MOESM2_ESM.docx]

**Supplemental Table 2: Comparison of baseline demographics and OCT findings between eyes that completed the 8-year follow-up and those lost to follow-up at any point before the 8-year mark.**

|  | **Followed (92 eyes)** | **Lost to Follow-up (184 eyes)** | **P-value** |
| --- | --- | --- | --- |
| **Age (mean ± SD)** | 73.9 ± 7.9 | 74.1 ± 8.0 | 0.85 |
| **Proportion female** | 55.3% (51/92) | 50.0% (92/184) | 0.444 |
| **Prevalence of MA** | 28.3% (26/92) | 31.0% (57/184) | 0.678 |
| **Presence of IRF** | 53.3% (49/92) | 60.3% (111/184) | 0.301 |
| **Presence of SRF** | 70.7% (65/92) | 71.2% (131/184) | 1.0 |
| **Presence of both (IRF and SRF)** | 33.7% (31/92) | 39.1% (72/184) | 0.429 |

MA, macula atrophy; IRF, intraretinal fluid; SRF, subretinal fluid
